# Multifunctional Hydrogels for Diabetic Wound Healing: Design Strategies and Microenvironmental Remodeling Mechanisms

**DOI:** 10.3390/gels12070640

**Published:** 2026-07-17

**Authors:** Yu Zeng, Yijun Huang, Xinying Zhong, Li Li, Dao Chen, Lin Li

**Affiliations:** 1 Department of Cell Biology, School of Preclinical Medicine, Zunyi Medical University, Zunyi 563000, China; 18385024288@163.com (Y.Z.); 18381813490@163.com (X.Z.); 18785384769@163.com (L.L.); chendao@zmu.edu.cn (D.C.); 2 The First Clinical College, Zunyi Medical University, Zunyi 563099, China; 13656951073@163.com

**Keywords:** diabetic wounds, multifunctional hydrogels, reactive oxygen species scavenging, immune modulation, clinical translation

## Abstract

Diabetic wounds remain a major clinical challenge owing to persistent dysregulation of the wound microenvironment, which substantially limits the effectiveness of conventional therapies. In recent years, multifunctional hydrogels have emerged as promising platforms for diabetic wound management, attributed to their excellent biocompatibility, tunable physicochemical properties, and unique capacity to actively remodel pathological microenvironments through integrated therapeutic functions. This comprehensive narrative review provides an in-depth synthesis of the pathogenesis and current therapeutic strategies for diabetic wounds, with a particular focus on recent advances in multifunctional hydrogels, as well as their classification, design principles, mechanisms of action, and translational potential. Furthermore, emerging directions are discussed as promising approaches for next-generation therapies, including intelligent closed-loop systems, interdisciplinary technological convergence, and the integration of bioactive components derived from traditional Chinese medicine. Collectively, these advances are poised to facilitate the transition from passive wound coverage to active microenvironment remodeling, paving the way for precision and personalized diabetic wound care.

## 1. Introduction

Diabetes is a prevalent chronic metabolic disorder that poses a substantial global health burden. In 2024, the number of adults with diabetes worldwide exceeded 500 million, and this figure is projected to rise to approximately 900 million by 2050 [1]. Among diabetic patients, the incidence of diabetic wounds reaches up to 25%, with a five-year recurrence rate of 66% [2]. Under persistent hyperglycemia, diabetic wound pathogenesis involves multiple interconnected factors, including vascular dysfunction and peripheral neuropathy, microenvironmental disruption, cellular dysfunction, growth factor imbalance, and infection [3,4]. The interplay of these factors impedes normal wound healing, ultimately resulting in chronic wounds characterized by delayed or impaired healing [5]. Diabetic foot ulcers, one of the most prevalent forms of diabetic wounds, represent a major public health concern due to their high incidence, recurrence rate, healing difficulty, and risk of amputation [6,7,8,9,10,11].

A variety of clinical strategies are currently employed for diabetic wound management, including surgical debridement, skin grafting, stem cell therapy, negative pressure wound therapy, hyperbaric oxygen therapy, and wound care management. Among these, wound dressings are widely used in clinical practice owing to their convenient clinical application, cost-effectiveness, and localized therapeutic effects [12]. Based on composition and functional characteristics, dressings can be broadly categorized into traditional and advanced types [13]. Conventional dressings—such as natural gauze, synthetic fiber dressings, and oil-based dressings—are economical and readily available, providing a basic physical barrier and exudate absorption [14]. Advanced dressings, including foam, film, hydrocolloid, nanofiber, and hydrogel-based systems, offer superior moisture retention, breathability, and antimicrobial activity, thereby promoting a moist wound healing environment [13]. Nevertheless, several challenges persist (as Table 1). For example, although silver-ion-loaded nanofiber dressings exhibit broad-spectrum antimicrobial effects, their long-term use raises cytotoxicity concerns [15]. Moreover, some advanced dressings are suitable only for specific clinical scenarios. For example, foam dressings possess high absorbency and are not suitable for dry or minimally exuding wounds, as they may cause excessive wound desiccation, thereby disrupting the moist healing environment and delaying the healing process [16]. Additionally, some dressings, such as Smart nanofiber dressings, face significant barriers to large-scale clinical translation due to their complex manufacturing processes and high economic costs [17].

Hydrogels have emerged as a promising class of wound dressings due to their unique hydrophilic three-dimensional network, which combines favorable moisture retention, biocompatibility, and high design flexibility [18]. Based on the volume of published literature and the depth of investigation, multifunctional hydrogels have become one of the most intensively investigated categories of advanced wound dressings for diabetic wound management [19,20]. Recent studies have demonstrated that stimulus-responsive hydrogels can dynamically respond to pathological cues within diabetic wounds. When combined with controlled drug delivery systems, these materials enable on-demand therapeutic release in response to changes in pH, glucose concentration, or Reactive Oxygen Species (ROS) levels [21]. Additionally, these hydrogels possess immunomodulatory properties, appropriately engineered to modulate macrophage polarization toward a pro-regenerative (anti-inflammatory, reparative) phenotype, thereby improving the local microenvironment [21]. Furthermore, through the synergistic delivery of three-dimensional biomimetic networks and bioactive molecules, hydrogels provide a favorable microenvironment for cell adhesion, proliferation, and migration [22]. Notably, these multifunctional capabilities are designed to target multiple pathological features of diabetic wounds, offering a promising platform for precise and synergistic repair (as shown in Table 2). Consequently, researchers are currently focusing on the optimized design of multifunctional hydrogel systems to achieve controlled drug release and intelligent responsiveness to the wound microenvironment.

This review summarizes recent advances in multifunctional hydrogels for diabetic wound treatment, focusing on their classification, design strategies, mechanisms of action, and clinical translation. Overall, this review establishes a pathology- and translation-oriented framework that shifts hydrogel design in diabetic wound management from isolated functional development toward an integrated approach driven by disease mechanisms and clinical needs.

**Table 1:** Advantages and disadvantages of traditional versus advanced wound dressings.

Category	Subcategory	Functional Positioning	Key Characteristics	Representative Examples	References
Traditional Dressings	Natural gauze	Passive barrier	Strong absorbability, soft texture, and low cost; however, prone to adherence, unable to maintain a moist environment, and exhibiting poor barrier function.	Cotton gauze, linen gauze	[23]
	Synthetic fiber	Passive barrier	Softer and more comfortable than natural gauze, with good absorbability; however, it is also prone to adhesion and cannot maintain a moist environment.	Viscose fiber dressing, polyester fiber dressing	[12,24]
	Oil-based dressing	Passive barrier	Provides isolation, prevents adhesion, and maintains wound moisture; however, it has poor breathability and does not absorb exudate.	Vaseline gauze, paraffin gauze	[23]
Advanced Dressings (Non-hydrogel)	Film dressing	Passive + Active transition	Transparent, adhesive, permeable to water vapor, and bacteria-proof, thereby facilitating wound observation; however, it has poor absorbability and is not suitable for wounds with heavy exudate.	Polyurethane film dressing	[23]
	Foam dressing	Microenvironment management	Possesses a porous structure and high absorbability, provides thermal insulation, and is soft and comfortable; however, it is opaque and may adhere to dry wounds.	Polyurethane foam dressing, polyvinyl alcohol foam dressing	[24]
	Hydrocolloid	Active moist healing	Forms a gel upon contact with wound exudate, provides a moist environment, and is non-adherent; however, it is not suitable for severely infected or highly exuding wounds.	Sodium carboxymethyl cellulose composite dressing	[24]
	Alginate dressing	Active moist healing + hemostasis	Exhibits extremely high absorbability and good hemostatic effect, and forms a gel via ion exchange with exudate; however, it requires a secondary dressing for fixation.	Calcium alginate fiber dressing	[13]
	Drug/Bioactive dressing	Active therapeutic intervention	Loaded with silver ions, growth factors, and antibiotics, it actively intervenes in the healing process; however, it has high cost and potential cytotoxicity.	Silver ion dressing, growth factor dressing	[15]
	Tissue-engineered skin	Active tissue regeneration	Contains living cells or acellular scaffolds and acts as a skin substitute that directly participates in tissue regeneration; however, it involves complex preparation and extremely high cost.	Fibroblast-containing dermal substitute, acellular dermal matrix	[25,26]

Hydrogels, as a major subcategory of advanced dressings, are discussed separately in the main text.

**Table 2:** Comparison of Wound Dressing Systems.

Comparison Dimension	Traditional Dressings (e.g., Gauze, Cotton Pads) [23]	Commercially Available Advanced Dressings (e.g., Foam, Hydrocolloid, Hydrogel) [23,24]	Emerging Multifunctional Hydrogels (Smart Responsive, Drug-Loaded, etc.) [27,28]
Primary Function	Passive coverage, exudate absorption, physical barrier	Maintenance of a moist wound environment, promotion of autolytic debridement, partial exudate management	Active modulation of the wound microenvironment and stimuli-responsive behavior to pathological microenvironmental cues
Mechanism of Action	Dry wound healing principle	Moist wound healing principle with limited functional specificity	Dynamic responsiveness (pH/temperature/enzyme/ROS) enabling on-demand release of therapeutic agents or bioactive factors
Main Materials	Cotton, linen, synthetic fibers	Polyurethane foam, hydrocolloids, alginates	Biomimetic polymer materials (e.g., gelatin, hyaluronic acid), nanocomposites, 3D-printed or engineered hydrogel systems
Mechanical Properties	Fixed mechanical strength; prone to adherence to wound beds; limited conformability	Improved flexibility and conformability; however, some films exhibit limited exudate absorption capacity	Tunable properties including high stretchability, self-healing ability, injectability, or sprayability for adaptation to dynamic wound environments
Antibacterial & Bioactive Properties	No intrinsic bioactivity; requires external medicated coatings	Some products incorporate antibacterial agents (e.g., silver ions) or growth factors, with limited release control	Integration of multifunctional properties including antibacterial, antioxidant, anti-inflammatory, and pro-angiogenic activities
Clinical Advantages	Low cost and wide availability; however, frequent dressing changes may cause secondary tissue damage	Reduced dressing frequency and improved patient comfort compared with traditional dressings	Potential to accelerate wound healing and reduce scarring; reported studies suggest improved healing outcomes (up to 30–40% in selected preclinical/clinical studies)
Major Limitations	Poor moisture retention and higher risk of infection and pain	Higher cost than traditional dressings; potential cytotoxicity concerns in some formulations	Translational barriers including scalable manufacturing, standardization, long-term safety evaluation, and regulatory approval
Market Status	Still widely used, particularly for superficial or clean wounds	Major segment of the global advanced wound care market, with continuous growth	Mostly in preclinical or early clinical stages; only a limited number of products have entered clinical translation or regulatory submission
Cost-Effectiveness	Low unit cost, but high dressing frequency and delayed healing may increase overall treatment cost	In certain clinical scenarios, improved healing efficiency can make them more cost-effective than traditional dressings	High research and development costs; cost-effectiveness depends on clinical validation of reduced complications and shortened hospital stays

Market data is sourced from a market report by Grand View Research.

## 2. Classification of Hydrogels and Optimization Strategies

**Figure 1:**
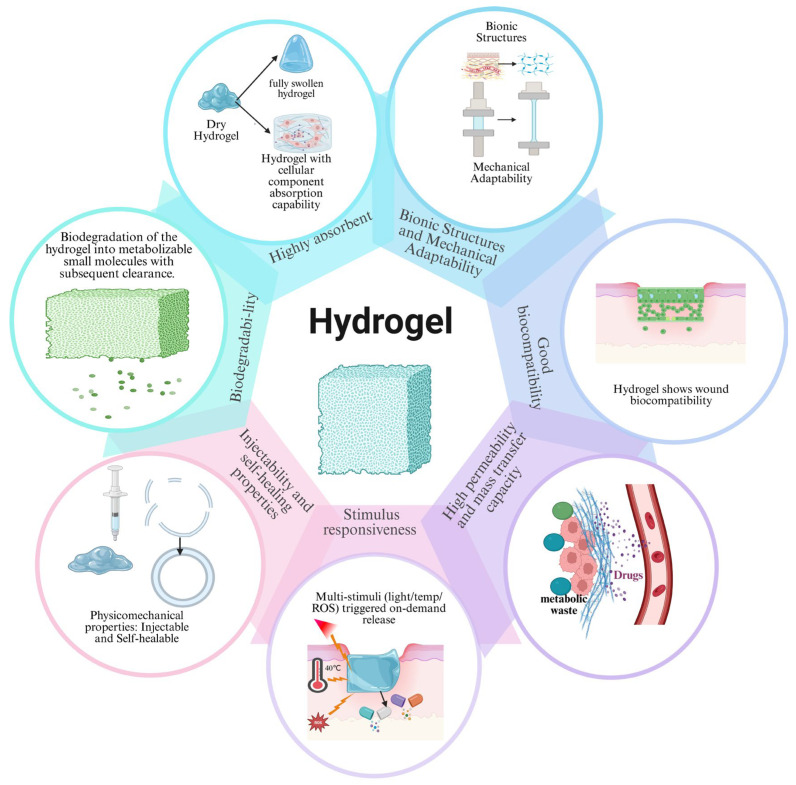
Functional characteristics of hydrogels in diabetic wound healing.

Hydrogels are highly hydrophilic polymeric networks capable of rapidly absorbing and retaining large volumes of fluid without dissolving [29]. Over the past several decades, significant advances in material science and bioengineering have greatly expanded both the diversity and functional complexity of hydrogel systems (as shown in Figure 1) [30]. Hydrogels are commonly classified according to three major criteria: cross-linking mechanisms, stimulus responsiveness, and material origin [31]. These classification strategies not only determine the physicochemical and biological properties of hydrogels but also provide the foundation for their rational design in biomedical applications. In the context of diabetic wound management, researchers have progressively shifted from conventional hydrogel systems toward multifunctional and smart hydrogels capable of simultaneously addressing infection, oxidative stress, inflammation, impaired angiogenesis, and tissue regeneration. Therefore, understanding hydrogel classifications and corresponding optimization strategies is essential for the development of next-generation wound dressings.

### 2.1. Classification of Hydrogels

#### 2.1.1. Classification by Cross-Linking Method

Hydrogels are generally classified as physically cross-linked or chemically cross-linked systems according to the nature of the interactions responsible for network formation [29]. Physically cross-linked hydrogels are formed through non-covalent interactions, including hydrogen bonding, ionic interactions, hydrophobic associations, and electrostatic forces [32]. These hydrogels are commonly fabricated from natural polymers such as gelatin, sodium alginate, and chitosan, exhibiting excellent biocompatibility and biodegradability. However, their relatively weak mechanical strength and limited structural stability may restrict their application in mechanically demanding environments [33,34].

In contrast, chemically cross-linked hydrogels form stable three-dimensional networks through covalent bonds generated by radical polymerization, click chemistry, functional group reactions, or radiation-induced cross-linking. Representative materials include polyacrylamide and poly (ethylene glycol) (PEG)-based hydrogels, which offer superior mechanical properties and structural tunability. However, the irreversible nature of covalent cross-linking often renders these hydrogels difficult to degrade under physiological conditions [35]. Nevertheless, residual cross-linking agents and reaction by-products may introduce potential cytotoxicity concerns, leading to inferior biocompatibility compared to their physically cross-linked counterparts, thereby affecting their translational potential [36].

To overcome the limitations of conventional cross-linking approaches, emerging strategies such as enzyme-mediated cross-linking and dynamic covalent bonding have attracted increasing attention. These approaches combine favorable biocompatibility with enhanced structural stability, providing new opportunities for the development of multifunctional biomedical hydrogels [36,37].

#### 2.1.2. Classification by Response System

Stimulus-responsive hydrogels possess the ability to undergo reversible changes in their physicochemical properties when exposed to specific environmental cues, thereby enabling adaptive therapeutic functions [38,39]. According to the type of triggering stimulus, these systems can be broadly classified as physically responsive hydrogels (e.g., light, temperature, magnetic field, and electrical stimulation), chemically responsive hydrogels (e.g., pH, glucose, and reactive oxygen species), and multi-responsive hydrogels that integrate multiple sensing mechanisms [38]. Beyond biochemical responsiveness, mechanically adaptive hydrogels can sense and respond to mechanical cues; for example, a dynamic disulfide bond-based injectable lubricative hydrogel (ILH) provides mechanical protection under static conditions and exhibits reversible shear-thinning behavior under stress, offering a new strategy for designing adaptive dressings for diabetic wounds at mechanically active sites [40].

Compared with conventional dressings, stimulus-responsive hydrogels offer unique advantages in diabetic wound treatment because they can dynamically respond to pathological changes within the wound microenvironment. Such responsiveness enables on-demand and spatiotemporally controlled therapeutic delivery, thereby improving treatment precision while minimizing unnecessary drug exposure [41,42]. In contrast, the dynamic and spatiotemporal variability of key wound microenvironmental signals—including pH, glucose, ROS, temperature, and exudate composition—presents a major obstacle for the rational design of stimuli-responsive hydrogels capable of adapting to the evolving healing process [43].

#### 2.1.3. Classification by Source of Material

Based on material composition, hydrogels can be categorized into natural, synthetic, and semi-synthetic systems [44].

Natural polymers, including chitosan, hyaluronic acid, collagen, and agarose, possess intrinsic biological activities that support cell adhesion, proliferation, and tissue regeneration. However, their relatively poor mechanical properties, rapid degradation rates, and batch-to-batch variability may limit large-scale clinical application [45].

Synthetic polymers such as poly (ethylene glycol) (PEG), poly (vinyl alcohol) (PVA), and poly(vinylpyrrolidone) (PVP) provide excellent structural controllability and reproducible physicochemical properties. Nevertheless, most synthetic polymers lack intrinsic bioactivity and therefore require functional modification to achieve desirable biological performance [31].

To overcome the limitations of both categories, semi-synthetic hydrogels have emerged as a promising alternative. Through chemical modification of natural polymers or hybridization with synthetic components, semi-synthetic systems combine favorable biocompatibility with tunable mechanical properties. Representative materials such as gelatin methacryloyl (GelMA) and modified hyaluronic acid (HA) derivatives have become widely used platforms in diabetic wound healing applications [31].

In addition to the above classifications, hydrogels can also be categorized according to their functional properties, including self-healing, adhesive, conductive, and optical functions, as well as by their mode of application, such as preformed and injectable systems [46,47]. Importantly, these classification categories are not mutually exclusive. Contemporary hydrogel systems often integrate multiple design principles through hybridization, copolymerization, or composite fabrication strategies. Such integration gives rise to composite hydrogels that combine distinct functional components within a unified system thereby enabling synergistic enhancements in biological activity, mechanical performance, and therapeutic efficacy [48].

### 2.2. Strategies for the Optimized Design of Hydrogels

Although conventional hydrogels provide a moist healing environment and structural support for tissue regeneration, their therapeutic efficacy remains insufficient for addressing the multifactorial pathophysiology of diabetic wounds. Consequently, recent research has focused on the development of advanced hydrogel systems through structural, functional, and manufacturing optimization strategies. The rational design of hydrogel dressings depends on optimizing key physicochemical properties, including porosity, swelling behavior, degradation kinetics, mechanical strength, and rheology, which together determine mass transport, structural stability, and adaptation to dynamic wound environments [49]. Proper tuning of these parameters enables effective exudate control, tissue regeneration support, and balanced degradation, thereby promoting microenvironmental homeostasis and coordinated repair in diabetic wounds.

#### 2.2.1. Structural Optimization

Structural optimization aims to improve the mechanical stability, adaptability, and durability of hydrogel networks, primarily through the refinement of cross-linking mechanisms and network architectures. One common approach involves refining cross-linking mechanisms. For example, incorporating multiple hydrogen-bonding interactions can enhance the robustness of physically cross-linked hydrogels, whereas integrating dynamic covalent bonds such as Schiff-base linkages can simultaneously improve mechanical strength and self-healing capacity [50,51]. In addition, double-network hydrogels, interpenetrating polymer networks, and supramolecular hydrogels have emerged as effective strategies for overcoming the mechanical limitations of traditional systems while maintaining favorable biocompatibility [52]. The practical advantages of these structural strategies depend on specific wound requirements. Physically cross-linked hydrogels provide excellent biocompatibility and injectability, dynamic covalent networks enable self-healing and adaptive behavior, while double-network and supramolecular systems enhance mechanical reinforcement and dynamic remodeling; however, these improvements may involve trade-offs, including limited mechanical stability, reduced molecular diffusion and cellular infiltration, and increased fabrication complexity [27,53]. Therefore, structural optimization should be tailored to wound location, mechanical demands, and healing stages to balance mechanical performance, biological functionality, and translational feasibility.

#### 2.2.2. Functional Optimization

Functional optimization focuses on enabling hydrogels to actively regulate the wound microenvironment rather than merely serving as passive dressings.

Stimulus-responsive hydrogels can sense pathological cues such as elevated glucose levels, excessive reactive ROS accumulation, and pH fluctuations, thereby triggering controlled therapeutic release [38,54,55]. This adaptive responsiveness improves treatment precision and allows therapeutic interventions to better match the dynamic progression of wound healing.

Furthermore, dynamic network architectures can endow hydrogels with self-healing behavior, shape-memory properties, and programmable drug-release profiles. These characteristics facilitate more effective adaptation to the spatiotemporal changes occurring during wound repair [56,57,58,59].

Recent studies have also highlighted the importance of immunomodulatory hydrogel systems capable of regulating macrophage polarization, reducing chronic inflammation, and promoting tissue regeneration, thereby addressing one of the key pathological barriers in diabetic wound healing [60,61].

#### 2.2.3. Biomimetic and Composite Design Strategies

Biomimetic design is another important direction in hydrogel development. By mimicking the composition, architecture, and mechanical characteristics of the native extracellular matrix (ECM), biomimetic hydrogels provide a favorable microenvironment for cellular adhesion, proliferation, migration, and differentiation [62,63].

Meanwhile, composite strategies that combine natural and synthetic polymers, nanomaterials, bioactive molecules, and conductive components have enabled the development of multifunctional hydrogel platforms with enhanced antibacterial, antioxidant, angiogenic, and tissue-regenerative properties [64].

Conductive hydrogels [65], injectable hydrogels [55], and Shape Memorable and Self-Healable Smart Hydrogels [66] have emerged as particularly promising platforms for diabetic wound management, reflecting the ongoing transition from single-function dressings toward integrated therapeutic systems.

Furthermore, integrating biomimetic design with 3D/4D printing technology allows hydrogels to transition from simple morphological adaptation to spatiotemporal functional programming [51,67].

#### 2.2.4. Manufacturing and Translational Optimization

Advanced manufacturing technologies have further accelerated the evolution of hydrogel-based wound dressings.

Among these technologies, 3D printing enables personalized fabrication of hydrogel architectures with precisely controlled geometry and porosity, while 4D printing introduces dynamic structural changes in response to environmental stimuli [68]. These approaches facilitate the development of patient-specific wound dressings with enhanced adaptability.

At the microscale, hydrogel-based delivery platforms have emerged as versatile systems for the localized and controlled delivery of diverse bioactive agents and therapeutic cargos, including growth factors, stem cells, extracellular vesicles, antimicrobial agents, nucleic acids, peptides, and small-molecule therapeutics [69,70,71]. By regulating the spatial and temporal release of these therapeutic cargos, hydrogel systems can enhance angiogenesis, modulate inflammatory responses, regulate immune responses, and promote tissue regeneration during diabetic wound healing. Among these delivery strategies, microspheres, nanoparticles, and exosome-loaded systems have been extensively integrated with hydrogel matrices to achieve controlled and sustained therapeutic release [72,73]. Such integration enables localized and prolonged therapeutic effects while minimizing systemic exposure and improving the efficacy of encapsulated cargos.

Despite these advances, several challenges remain before widespread clinical translation can be achieved, including manufacturing scalability, long-term biosafety, quality control, storage stability, and regulatory approval. Addressing these issues will be critical for the successful implementation of multifunctional hydrogel technologies in diabetic wound management.

Owing to their highly hydrated three-dimensional networks, hydrogels provide exudate management, mechanical adaptability, biocompatibility, molecular transport, stimulus responsiveness, injectability, self-healing capability, and biodegradability. These integrated properties enable hydrogels to modulate the wound microenvironment and support tissue repair in diabetic wounds.

## 3. The Role of Multifunctional Hydrogels in Modulating the Microenvironment of Diabetic Wounds

**Figure 2:**
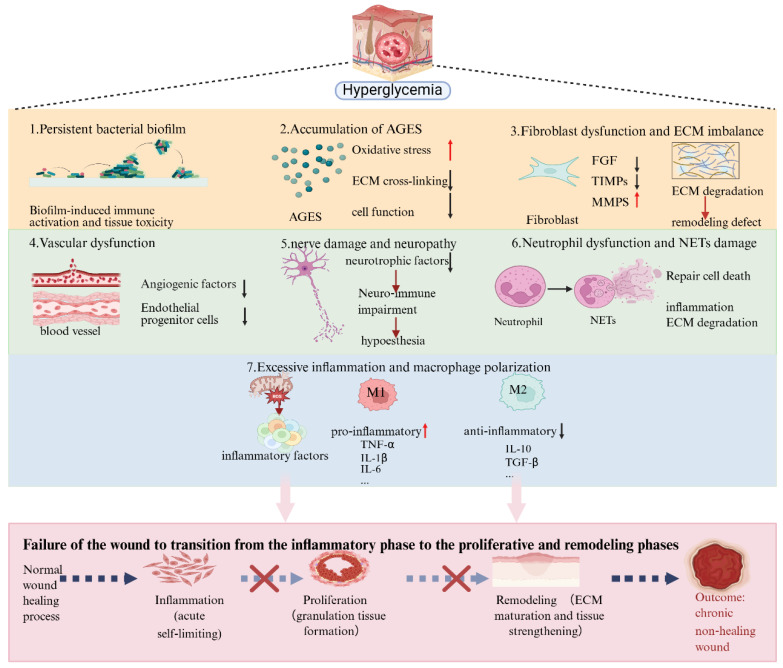
Schematic illustration of the pathological microenvironment in diabetic wounds.

Diabetic wounds are characterized by a complex pathological microenvironment involving persistent hyperglycemia, oxidative stress, dysregulation of the immune microenvironment, chronic inflammation, microbial infection, impaired angiogenesis, ECM remodeling disorders, and cellular senescence, as shown in Figure 2 [74,75,76,77].

Persistent hyperglycemia triggers excessive ROS generation, chronic inflammation, abnormal pH conditions, and bacterial infection, resulting in macrophage polarization imbalance, impaired growth factor signaling, vascular dysfunction, peripheral neuropathy, and defective tissue repair. The synergistic effects of these pathological alterations ultimately lead to chronic, non-healing wounds.

Persistent hyperglycemia, a key pathological driver, induces immune cell dysfunction, including an imbalance in classically activated (M1) macrophage and alternatively activated (M2) macrophage polarization, and dysregulation of neutrophil extracellular trap formation (NETosis), thereby prolonging inflammation and suppressing tissue repair [78,79]. High glucose levels also promote excessive mitochondrial ROS production, exacerbating oxidative stress and further amplifying inflammatory signaling [80]. Additionally, hyperglycemia-induced sensory deficits delay injury detection, increasing the risk of infection [81,82], while elevated glucose levels provide nutrients for pathogenic microorganisms, facilitating biofilm formation and bacterial phenotypic shifts [82,83]. At the molecular level, sustained overexpression of pro-inflammatory cytokines, including Tumor Necrosis Factor-alpha (TNF-α), Interleukin-1 beta (IL-1β), and Interleukin-6 (IL-6), activates pathways such as Nuclear Factor kappa-light-chain-enhancer of activated B cells (NF-κB), establishing a positive feedback loop of inflammation while suppressing anti-inflammatory factors (e.g., IL-10) [84,85]. Growth factors (GF), essential mediators of tissue regeneration, exhibit severely impaired bioavailability and signaling activity under hyperglycemic conditions [86]. Local physicochemical factors, including pH [87] and humidity [88], further modulate these pathological processes, collectively destabilizing the wound microenvironment.

The interplay among these mechanisms and the resulting vicious cycle renders conventional hydrogels ineffective in reversing the pathological process. To overcome these limitations, recent advances in multifunctional hydrogel engineering have enabled the development of smart therapeutic platforms capable of simultaneously modulating multiple pathological processes through microenvironment-responsive and bioactive strategies [89]. Multifunctional hydrogels can be designed via structural optimization (e.g., dynamic networks, biomimetic architectures) and functional integration (e.g., drug delivery, incorporation of nanomaterials, and stimuli-responsive elements), allowing coordinated regulation of the diabetic wound microenvironment.

Building on the discussion above, the following sections will systematically delineate the specific mechanisms through which multifunctional hydrogels facilitate diabetic wound healing.

### 3.1. Metabolic Regulation: Targeting Hyperglycemia and Cellular Senescence

Hyperglycemia is a fundamental pathological driver of diabetic wound progression. Sustained elevations in glucose levels induce oxidative stress, advanced glycation end-product (AGE) accumulation, endothelial dysfunction, peripheral neuropathy, cellular senescence, and chronic inflammation, collectively impairing tissue regeneration and wound closure [90,91].

#### 3.1.1. Classification of Hydrogel-Based Metabolic Regulation Strategies

Current hydrogel-based metabolic regulation strategies can be broadly categorized into three classes: (i) direct glucose consumption and microenvironment remodeling, (ii) localized delivery of hypoglycemic agents, and (iii) suppression of hyperglycemia-induced cellular senescence.

#### 3.1.2. Representative Strategies and Examples

Microenvironment remodeling: The multifunctional COH-GB hydrogel developed by Ma [64] represents a typical microenvironment-remodeling strategy. By simultaneously reducing local glucose concentration, alleviating tissue hypoxia, scavenging ROS, and regulating pH, the hydrogel reconstructs the diabetic wound microenvironment. Moreover, glucose-responsive nitric oxide (NO) release enables simultaneous antibacterial and angiogenic activities, demonstrating the advantages of integrated metabolic regulation.

Localized drug delivery: Localized drug delivery represents another promising strategy. Zhou [54] incorporated insulin into an AP/SA hydrogel, achieving sustained local glucose reduction and prolonged therapeutic activity. Compared with conventional administration, hydrogel-mediated delivery provides more stable glucose control while simultaneously enhancing cell proliferation and angiogenesis.

Anti-senescence approach: In addition to regulating glucose levels directly, recent studies have increasingly focused on hyperglycemia-induced cellular senescence. The GelMA/PNS/Alg@IGF-1 hydrogel developed by Wang [92] delivers insulin-like growth factor-1 (IGF-1) to counteract cellular senescence while simultaneously reducing oxidative stress, thereby restoring cellular function and promoting vascular regeneration.

In short, these strategies demonstrate that effective metabolic regulation extends beyond glucose reduction alone and includes the prevention of senescence-associated dysfunction and restoration of cellular homeostasis.

In summary, metabolic regulation in diabetic wound healing extends beyond glucose control to include senescence modulation and cellular homeostasis restoration, yet its clinical translation is limited by poor physicochemical stability and insufficient spatiotemporal responsiveness to dynamic wound microenvironment signals [77,93,94]. Therefore, integrating metabolic modulation with antioxidant, anti-inflammatory, and pro-angiogenic functions within multifunctional hydrogel platforms is essential to address the multifactorial pathophysiology of diabetic wounds.

### 3.2. Regulation of the Immune Microenvironment

Persistent inflammation driven by an imbalance in macrophage polarization and excessive neutrophil activation is a major obstacle to diabetic wound healing. High glucose levels sustain M1-type macrophage activation while hindering M2-type polarization, which leads to prolonged inflammation, defective tissue repair, and eventual wound chronicity [95,96,97]. To counteract these immune disturbances, researchers have developed multifunctional hydrogels that can steer macrophage polarization, fine-tune inflammatory signaling pathways, limit NETosis, and restore cytokine balance.

#### 3.2.1. Macrophage Repolarization

Hydrogels that encourage M2 polarization primarily work by restoring oxidative metabolism in macrophages, suppressing pro-inflammatory gene expression, and enhancing repair-related gene programs. For instance, the Ac2-26-loaded hydrogel reprograms macrophages via FPR2/PI3K/AKT signaling, which suppresses TLR-driven inflammation and restores oxidative phosphorylation to shift the diabetic wound niche from pro-inflammatory to pro-regenerative [98]. In another study, the SilMA-FGF21/CoS hydrogel not only promotes M2 polarization but also boosts angiogenesis through Janus kinase/signal transducer and activator of transcription (JAK/STAT)-mediated upregulation of vascular endothelial growth factor (VEGF), tackling both immune dysfunction and poor vascularization [99]. Natural polyphenol-based materials, such as protocatechuic aldehyde hydrogels, remodel the immune microenvironment by scavenging ROS, activating nuclear factor erythroid 2-related factor 2 (Nrf2), and suppressing NF-κB signaling—all without introducing external biological agents [100]. Transcriptomic analyses further show that the AP@HA-Si InjGel enhances the expression of reparative markers like arginase 1 (Arg1) and mannose receptor, C type 1 (Mrc1), confirming the molecular basis of macrophage reprogramming [101].

#### 3.2.2. Modulation of Inflammatory Signaling Pathways

The coordinated regulation of multiple signaling pathways is central to immune modulation in diabetic wound healing. Impaired activation of the hypoxia-inducible factor-1 (HIF-1) pathway has been identified as a major cause of persistent wound non-healing, whereas activation of this pathway effectively promotes tissue repair [102,103,104]. Similarly, activation of the Nrf2 pathway [105] and inhibition of the NF-κB pathway [106] have been shown to markedly accelerate healing. These observations indicate that these signaling pathways represent promising therapeutic targets. The GelMA-FICZ hydrogel accelerates diabetic wound healing by activating AhR to restore impaired PINK1/Parkin-mediated mitophagy, thereby blocking mtDNA leakage and subsequent cGAS-STING/NLRP3 inflammasome activation, which reverses M1-dominant inflammation and promotes M2 polarization. [107]. The 4-hydroxychalcone (4HC) hydrogel developed by Li [108] promotes M2 macrophage polarization by inhibiting the TLR/IL-17/TNF signaling axis, thereby facilitating the wound transition from the inflammatory to the proliferative phase.

#### 3.2.3. Cytokine Regulation and NETosis Control

Dysregulated cytokine expression and excessive NET formation further exacerbate chronic inflammation in diabetic wounds. Persistent elevation of TNF-α, IL-1β, and related cytokines directly impairs healing [109]. Hydrogel interventions such as quercetin nanoparticle-chitosan/fibronectin (QnChS) scaffolds reduce TNF-α and IL-1β levels while upregulating basic fibroblast growth factor (bFGF), achieving combined anti-inflammatory and regenerative effects [110]. Another study reported that the mPDA-PEI@GelMA micro-cage scavenges NETs via a non-contact electrostatic capture mechanism, confining cationic nanoparticles within GelMA microspheres to safely immobilize cfDNA from wound exudate, thereby alleviating NETs-driven inflammation and accelerating healing, as demonstrated in STZ-induced diabetic mice and human neutrophil models [111]. This NETs-scavenging capability represents a promising strategy for mitigating chronic inflammation and promoting wound repair.

Collectively, immunomodulatory hydrogels with promising translational relevance primarily exert their effects through macrophage M1–M2 polarization and reactive oxygen species (ROS) scavenging [112]. However, most of these strategies remain at the stage of mechanistic studies or preclinical animal models, with limited clinical validation (as shown in Table 3). Emerging approaches, including macrophage repolarization, NETosis regulation, multi-targeted hydrogel interventions, and signaling pathway modulation, underscore the pivotal role of immune microenvironment remodeling in accelerating diabetic wound healing, while highlighting the necessity of advancing these strategies toward clinical translation.

### 3.3. Scavenging ROS to Alleviate Oxidative Stress

Persistent oxidative stress induced by hyperglycemia represents a critical pathological mechanism underlying impaired wound healing in diabetes [80,113]. While moderate levels of reactive oxygen species (ROS) contribute to host defense, excessive accumulation induces cellular oxidative damage, DNA lesions, and pro-inflammatory factor release, thereby disrupting vascular endothelial function and impairing angiogenesis, which hinders wound healing [114]. At the molecular level, excessive ROS accumulation upregulates *microRNA-200c* (*miR-200c*) expression, mediating apoptosis and cellular senescence, whereas *miR-200c* inhibition enhances wound healing [115]. Therefore, modulation of ROS levels is a pivotal strategy for promoting diabetic wound healing.

#### Classification and Representative Examples of ROS-Modulating Hydrogels

In recent years, various ROS-modulating hydrogels have been developed and can be classified into three categories based on their mechanisms of action: (i) direct ROS scavengers, (ii) synergistic combination of direct ROS scavenging and source inhibition and (iii) systems that act synergistically with other reparative factors.

Direct ROS scavengers: The fullerene/sodium alginate composite hydrogel (6HPB@C60) developed by Wang [113] leverages the potent antioxidant properties of fullerene to markedly reduce ROS levels at the wound site and promote healing. Synergistic combination of direct ROS scavenging and source inhibition: The ORH hydrogel developed by Lee [116] exerts dual-mode ROS regulation by directly scavenging reactive oxygen species via catalase while simultaneously alleviating hypoxia through sustained oxygenation to curb ROS overproduction, thereby breaking the oxidative-inflammatory cycle and accelerating diabetic wound healing. Synergistic antioxidant-regenerative systems: The TA/PDGF co-delivery hydrogel developed by Kim [117] integrates tannic acid-mediated ROS elimination with platelet-derived growth factor (PDGF)-driven tissue regeneration, achieving a synergistic effect between “damage clearance” and “active repair.”

In summary, direct ROS scavenging provides rapid but consumptive neutralization, source-inhibiting systems enable on-demand intervention via ROS-triggered drug release, and synergistic platforms integrate multi-target functions to comprehensively reshape the inflammatory microenvironment—representing a logical evolution from passive supplementation to intelligent regulation and ultimately to integrated combination therapy.

### 3.4. Promoting Neurovascular Regeneration and Functional Recovery

#### 3.4.1. Pathological Basis of Neurovascular Dysfunction

During the progression of diabetes, persistent hyperglycemia can induce peripheral neuropathy, manifesting as either reduced sensation or abnormal sensation [81]. Reduced sensation impairs the ability to detect injury, delaying timely intervention, whereas abnormal sensation, such as hyperalgesia, negatively affects treatment adherence. Together, these factors form a vicious cycle that hinders wound healing [118]. Concurrently, hyperglycemia-induced vascular lesions result in inadequate tissue perfusion, exacerbate ischemia and hypoxia, and consequently delay wound repair [119,120,121]. Therefore, promoting neurovascular regeneration and restoring functional integrity is critical for achieving complete wound healing.

#### 3.4.2. Hydrogel-Based Strategies for Neurovascular Regeneration

To address these challenges, researchers have explored diverse design strategies for multifunctional hydrogels. One approach focuses on the delivery of bioactive factors; Xiong [122] developed a hydrogel that the hydrogel establishes a neurogenesis-angiogenesis crosstalk by activating Mg^2+^-triggered chemokine signaling (IL-8/CCL5) to recruit BMSCs, while G-sEVs deliver pro-neural cues that induce neurogenic differentiation and reprogram macrophages toward the M2 phenotype, collectively initiating a self-sustaining regenerative cycle that amplifies vascularization and tissue repair. In contrast, Kim [58] developed the AFGKLT hydrogel activates integrin-FAK mechanosignaling via aligned fibers to drive M2 macrophage polarization and Schwann cell maturation, while KLT peptide upregulates VEGF/PDGF through VEGFR, synergistically amplifying IL-10/VEGF/BDNF paracrine loops to establish a self-sustaining immune-angiogenic-neurogenic axis, ultimately accelerating diabetic wound closure with functional neovascularization and neurogenesis. The PABC hydrogel promotes diabetic wound healing through Cu^2+^-mediated activation of the HIF-1α/VEGF signaling axis in endothelial progenitor cells, thereby enhancing angiogenesis and collagen deposition. Remarkably, this pro-healing efficacy is achieved without the incorporation of any exogenous growth factors or bioactive molecules, underscoring its unique advantage over conventional growth factor-based therapies [123].

#### 3.4.3. Emerging Targets: ECM Remodeling

Beyond vascular and neural repair, ECM remodeling has emerged as an increasingly important therapeutic target. Hyperglycemia-induced upregulation of matrix metalloproteinases (MMPs) and disruption of the MMP/tissue inhibitors of metalloproteinases (TIMP) balance accelerate ECM degradation, impairing tissue regeneration [124]. Therefore, hydrogels capable of restoring ECM homeostasis may significantly improve long-term healing outcomes. In diabetic wounds, MMP-9-responsive hydrogels—such as those incorporating metal-chelating dipeptides (e.g., L-carnosine) or anti-inflammatory agents (e.g., celecoxib)—effectively suppress MMP-9 activity, thereby mitigating excessive extracellular matrix degradation and accelerating wound healing [125].

Collectively, these strategies—ranging from bioactive factor delivery and biomimetic design to material intrinsic properties and ECM remodeling—provide a comprehensive framework for promoting neurovascular regeneration and tissue remodeling in diabetic wounds. Future designs may combine multiple mechanisms to achieve synergistic therapeutic benefits.

### 3.5. Antimicrobial and Anti-Biofilm Regulation

#### 3.5.1. Pathological Basis and Challenges of Infection in Diabetic Wounds

Diabetic wounds are highly susceptible to infection due to multiple factors. Localized hyperglycemia disrupts the skin barrier and induces dysbiosis of the cutaneous microflora, thereby creating a microenvironment favorable for pathogen colonization [82,83]. Hyperglycemia not only supplies abundant nutrients for microbial proliferation but also promotes biofilm formation and bacterial phenotypic transformation, thereby complicating infection management. Among pathogens, Staphylococcus aureus is the most prevalent due to its adaptability to hyperglycemic environments and strong adhesion properties [126,127]. Biofilms protect microorganisms from antibiotics and host immune responses, further increasing treatment difficulty and promoting antimicrobial resistance. Conventional antibiotic therapy is often ineffective in the long term, as prolonged use readily induces resistant strains [82,128,129]. Conventional management primarily relies on systemic or topical antibiotics [130]; however, the chronic and refractory nature of diabetic wounds renders prolonged antibiotic use prone to inducing resistant strains. Currently, multidrug-resistant bacteria represent a major global public health challenge [131]. Therefore, the development of dressings that combine antimicrobial and pro-healing properties is a key strategy for addressing this challenge.

#### 3.5.2. Representative Antimicrobial Hydrogel Systems

To address these challenges, multifunctional hydrogels have evolved from simple antimicrobial dressings toward more integrated antibacterial platforms. For instance, the PSE-AgNPs-PVA hydrogel [132] exerts broad-spectrum antibacterial activity through nano Ag^+^-mediated multi-target chemical damage, including bacterial membrane disruption, metabolic inhibition, and oxidative stress induction, effectively targeting *E. coli*, *S. aureus*, *K. pneumoniae*, and carbapenem-resistant *A. baumannii* (CRAB). In addition, it significantly enhances wound healing and tissue regeneration in both in vitro assays and a full-thickness skin defect mouse model. However, Ag^+^ exhibits potential cytotoxicity, and its long-term safety profile has not been fully evaluated, representing a major limitation for further clinical translation [133]. Another approach relies on physical membrane disruption without inducing drug resistance; In a MRSA-infected rat full-thickness wound model, the PVA/agarose double-network hydrogel incorporating hyperbranched polylysine (HBPL) and tannic acid (TA) achieves near-complete bacterial clearance through HBPL-mediated electrostatic membrane disruption, while also significantly accelerating wound healing and suppressing hypertrophic scar formation [134]. Natural compounds also offer promising alternatives. Baicalin-loaded hydrogels, for example, have demonstrated potent inhibitory effects against drug-resistant *Pseudomonas aeruginosa* (*P. aeruginosa*) [135]. The BPSFs@H hydrogel [8] employs black phosphorus nano-snowflakes (BPSFs) to generate ROS (^1^O_2_ and H_2_O_2_) via combined photothermal and photodynamic effects under near-infrared (NIR) irradiation, achieving 92.5% elimination of MRSA through oxidative damage. This target-independent physical–chemical synergistic strategy may help circumvent antibiotic resistance. However, its clinical translation is limited by the shallow tissue penetration of NIR light and the potential cytotoxicity associated with excessive ROS generation, which requires careful optimization in future designs. AMP-loaded hydrogels achieve antibacterial effects via charge-mediated selective membrane disruption, significantly reducing bacterial burden in STZ-induced diabetic mice and accelerating wound closure, while promoting re-epithelialization, angiogenesis, and collagen deposition; these effects are accompanied by reduced M1 macrophage polarization and ROS levels, but are limited by cytotoxicity at high concentrations, instability, and high production costs.

#### 3.5.3. Anti-Biofilm and Antifungal Strategies

Beyond planktonic bacteria, biofilm-associated infections require specialized approaches. The HG1MB1 hydrogel [136] leverages photodynamic therapy with enhanced photosensitizer penetration and ROS-mediated degradation of extracellular polymers, providing an innovative strategy to eradicate persistent biofilms. Additionally, antimicrobial strategies have been extended to mycology; multifunctional hydrogels may also be effective against multidrug-resistant fungal infections [137].

Taken together, these antimicrobial hydrogel strategies—ranging from broad-spectrum chemical agents and physical membrane disruption to natural products, photodynamic therapy, and antifungal approaches—provide complementary mechanisms to combat infection while promoting wound healing, thereby addressing a crucial aspect of diabetic wound management.

### 3.6. Regulation of Other Aspects of the Wound

#### 3.6.1. Regulation of Local pH

During diabetic wound healing, the wound microenvironment exhibits dynamic pH fluctuations: it is generally alkaline during the inflammatory or infectious phases and shifts toward a slightly acidic state during the reparative phase [138,139]. Such pH fluctuations directly affect the release kinetics of various growth factors, thereby differentially modulating the wound repair process [87]. Consequently, pH is recognized as a critical pathophysiological indicator and a regulatory target for diabetic wound healing [140]. In vitro and in vivo studies have demonstrated that maintaining a locally acidic environment promotes fibroblast proliferation and migration, epithelial regeneration, and collagen synthesis [141,142]. This effect is attributed to enhanced binding of growth factor receptors [87].

Leveraging these mechanisms, hydrogels have been engineered to actively regulate local pH. One category employs pH-responsive materials to enable visual color monitoring, thereby establishing an integrated “diagnosis and treatment” platform [143]. Another category consists of closed-loop systems with autonomous regulatory functions. For example, the OSA-GEL@GC hydrogel employs glucose oxidase (GOx) to convert endogenous glucose into gluconic acid, thereby achieving sustained local acidification [21].

#### 3.6.2. Modulation of Growth Factor Activity

Growth factors play central roles in orchestrating wound healing, yet their bioavailability and signaling efficacy are severely compromised in the diabetic microenvironment due to excessive proteolytic degradation, oxidative stress, and glycation [124]. Multifunctional hydrogels address these limitations through several strategies. First, they serve as protective reservoirs, encapsulating growth factors (e.g., EGF, FGF, PDGF, VEGF) within their three-dimensional network to shield them from enzymatic degradation and enable sustained release [97]. Second, hydrogel matrices can be engineered to present growth factors in a spatially controlled manner, mimicking the natural extracellular matrix and promoting cell-specific responses [144]. Third, stimuli-responsive hydrogels achieve on-demand delivery of growth factors precisely when and where they are needed [99]. For instance, VEGF-loaded MMP-responsive hydrogels have been shown to enhance angiogenesis in diabetic wounds by releasing the growth factor in response to locally elevated MMP activity [124]. In summary, these hydrogel-based strategies restore growth factor function, thereby accelerating tissue repair and regeneration.

#### 3.6.3. Prevention of Pathological Scarring

Inhibition of pathological scar formation constitutes another crucial aspect of functional wound healing. While normal repair involves physiological scarring, factors such as trauma, mechanical tension, or infection can disrupt this process [145]. Persistent inflammation drives excessive fibroblast activation via the TGF-β pathway, inducing aberrant collagen deposition and leading to hypertrophic scars or keloids [145,146,147].

To counteract these pathological mechanisms, hydrogels provide unique anti-scarring strategies, which can be broadly classified into two categories. The first strategy reduces wound tension through mechanical modulation, exemplified by HTA hydrogels that provide a tension-shielding barrier to protect the wound from mechanical stress [148]. The second strategy integrates multifunctional platforms, including TGF-β-targeting LA peptide hydrogels [149], PLGA/ZnO microneedle systems that combine antimicrobial, pro-healing, and anti-scarring properties [150], hydrogels loaded with asiaticoside or tannic acid [151,152], and smart responsive VP hydrogels [153]. Together, these strategies mitigate pathological scarring through distinct mechanisms, offering a multifaceted approach to achieve functional, aesthetically favorable wound healing.

In conclusion, regulating pH, modulating growth factors, and inhibiting scarring represent complementary strategies that jointly target the physicochemical microenvironment, molecular signaling, and long-term functional outcomes, each via distinct mechanisms.

**Figure 3:**
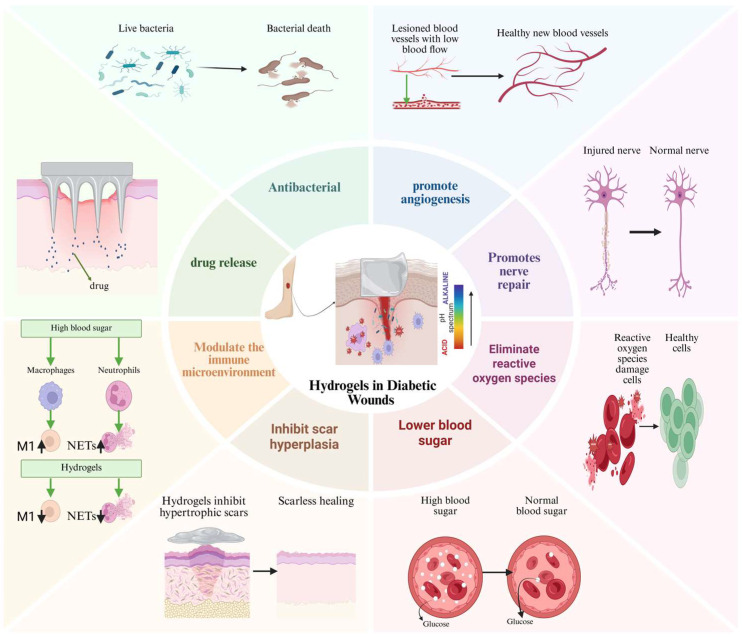
The role of multifunctional hydrogels in diabetic wounds. The green downward arrows indicate the next step in the process, the black upward arrows indicate an increase, the black downward arrows indicate a decrease, and the black horizontal arrows represent the outcomes of hydrogel treatment on the wound.

In summary (as shown in Figure 3), multifunctional hydrogels are increasingly engineered to modulate the diabetic wound microenvironment, restoring its homeostasis and thereby accelerating wound healing. A large body of in vitro and in vivo studies has demonstrated their promising potential in diabetic wound treatment, and emerging clinical studies have provided preliminary validation of these findings (as Table 3). However, the translation of these research advances into clinical applications still faces multiple challenges, including large-scale manufacturing, long-term stability, standardized evaluation methods, safety assessment, and regulatory barriers associated with multi-component systems [154]. For example, stimuli-responsive hydrogels suffer from limited stability under physiological conditions, complex fabrication processes, and insufficient clinical evidence [155]. In addition, metal-ion–loaded systems (e.g., silver ions) still raise concerns regarding potential cytotoxicity and unclear long-term biosafety [156]. Moreover, most hydrogel systems generally face a trade-off between sensitivity and stability. Collectively, these factors continue to hinder the clinical application and widespread translation of multifunctional hydrogels.

Multifunctional hydrogels not only possess the core functions of traditional hydro gels (water absorption and moisture retention) but also exhibit the following additional capabilities: (1) exerting antibacterial activity; (2) promoting angiogenesis; (3) promoting nerve repair; (4) scavenging reactive oxygen species; (5) lowering blood glucose; (6) inhib iting scar hyperplasia; (7) regulating the immune microenvironment; (8) enabling con trolled drug delivery. These functions directly address the complex pathophysiological mechanisms underlying diabetic wounds.

**Table 3:** Representative Multifunctional Hydrogels for Diabetic Wound Healing: Composition, Mechanisms, and Functional Outcomes.

Hydrogel System	Composition/Platform	Key Therapeutic Strategy	Mechanism (Condensed)	Main Functions/Outcomes	Study Type	Ref.
COH-GB gel	CMCS/OSA + GOx/Hb nanoflowers + HU	Glucose-triggered enzymatic cascade → NO release	GOx-generated H_2_O_2_ + Hb peroxidase-like activity → HU activation → NO production	Antibacterial, anti-inflammatory, angiogenesis; promotes collagen deposition	In vitro + animal models	[64]
AP/SA gel	AP-gel + SA + insulin	Glucose-responsive insulin release	Phenylboronic ester cleavage → insulin release → PI3K/Akt activation	Glycemic control, enhanced angiogenesis & proliferation	animal models	[54]
GelMA/PNS/Alg@IGF-1	GelMA + PNS + Alg + IGF-1	Sustained IGF-1 + herbal synergy	NF-κB inhibition + oxidative stress suppression → endothelial recovery + anti-senescence	Improved granulation tissue, angiogenesis, ECM remodeling	In vitro + animal models	[92]
HA-based self-healing gel	HA + β-CD + PVA + PEG + Ac2-26	Immune modulation	FPR2/PI3K/Akt activation + TLR inhibition → M1 → M2 shift	Anti-inflammatory, ECM regeneration, oxidative phosphorylation restoration	In vitro + animal models	[98]
SilMA-FGF21/CoS	Silk MA + CoS NPs + FGF21	Phase-dependent release system	H_2_S (inflammation phase) + FGF21 (proliferation phase) → JAK/STAT/VEGF	M2 polarization, antioxidant defense, enhanced angiogenesis	animal models	[99]
Natural collagen gel	Collagen + protocatechuic aldehyde	Intrinsic immunomodulation	ROS scavenging + macrophage reprogramming	Accelerated wound closure, antibacterial effect	animal models	[100]
AP@HA-Si InjGel	HA + silanol + arginine + puerarin	Neurovascular coupling	M2 polarization + ROS scavenging → angiogenesis + ECM remodeling	Enhanced tissue regeneration, scRNA-seq validated macrophage shift	animal models	[101]
GelMA-FICZ	GelMA + FICZ	AhR-mediated mitochondrial repair	AhR activation → PINK1/Parkin autophagy → cGAS-STING inhibition	Reduced inflammation, restored mitochondrial homeostasis	animal models	[107]
P-4HC nanofiber gel	Nanofiber + 4HC	Anti-inflammatory signaling blockade	TLR9/IL-17/TNF inhibition → M1 → M2 shift	Anti-inflammatory, M2 polarization, angiogenesis, improved chronic diabetic wound healing	animal models	[108]
QnChS scaffold	Chitosan + silk fibroin + quercetin	NF-κB inhibition	Downregulates TNF-α/IL-1β; upregulates VEGF/bFGF	Enhanced angiogenesis & collagen deposition	animal models	[110]
mPDA-PEI@GelMA	GelMA + mPDA-PEI microspheres	NETs scavenging system	Electrostatic cfDNA adsorption → TLR9 inhibition	Breaks NETs-inflammation loop; rapid healing	animal models	[111]
6HPB@C60 gel	SA + 6HPB@C60 complex	ROS scavenging + signaling activation	SOD-like activity + M1 → M2 shift + Ca^2+^/Wnt signaling	Promotes proliferation, angiogenesis, near-complete closure	animal models	[113]
ORH hydrogel	PPZ + CaO_2_ + CSCAT	Oxygen release + ROS clearance	Hypoxia reversal + enzymatic ROS elimination	Restores fibroblast function, accelerates healing	animal models	[116]
TSP–TP gel	PPZ + TA + PDGF	Antioxidant + growth factor release	TA scavenges ROS + PDGF activates angiogenesis	M2 polarization, vascular regeneration	animal models	[117]
AFGKLT hydrogel	Fibrin + VEGF-mimetic peptide	Neurovascular guidance	Integrin-FAK + VEGF signaling activation	Enhanced nerve–vessel–ECM regeneration	animal models	[58]
HA-ADH/OSA@Mg@sEVs	HA-ADH + OSA + Mg^2+^ + sEVs	Neurovascular feedback loop	Mg^2+^ recruitment + sEV-mediated neural differentiation	Neurovascular regeneration coupling	animal models	[122]
PABC hydrogel	PEGDA + ALG + BGNC	Ion-mediated regeneration	Cu^2+^/SiO_4_^4−^ release → HIF-1α/VEGF activation	Strong antibacterial + angiogenesis	animal models	[123]
PSE-AgNPs-PVA	PVA + AgNPs	Metal ion antibacterial	Ag^+^ membrane/protein/DNA disruption	Broad-spectrum antibacterial effect	animal models	[132]
PAHT hydrogel	PVA + agarose + TA + HBPL	ROS + membrane disruption	TA antioxidant + HBPL antibacterial	Anti-inflammatory, anti-scar, rapid healing	animal models	[134]
BPSFs@H hydrogel	PVA/Alg + black phosphorus	Photothermal therapy	NIR → heat + ROS generation	Biofilm destruction, angiogenesis	animal models	[157]
HG1MB1 gel	Gelatin + methylene blue	Photodynamic therapy	630 nm activation → ROS-mediated biofilm destruction	Strong antibacterial/antifungal effect	In vitro + animal models	[136]
CHR-COP gel	Small-molecule assembly	Cell wall stress targeting	MAPK/CWI pathway disruption in fungi	MDR Candida auris inhibition	animal models	[137]
OSA-GEL@GC	OSA + GOx + CAT	Feedback microenvironment control	Glucose → acid loop + ROS detoxification	pH/glucose homeostasis, enhanced healing	animal models	[21]
GAG-peptide gel	Peptide + sulfated GAGs	ECM-mimetic assembly	Electrostatic self-assembly + growth factor binding	Stem cell expansion, ECM-like mechanics	animal models	[144]
HTA hydrogel	HA + TA-Ag NPs	Shape-fixing + anti-fibrotic	Mechanotransduction (FAK/MCP-1) inhibition + photothermal effect	Scarless healing, antibacterial activity	animal models	[148]
LA-peptide gel	FXIIIa + LA peptides	TGF-β neutralization	TGF-β/Smad inhibition → reduced fibrosis	Anti-hypertrophic scar formation	animal models	[149]
MN-C/P-Z patch	Microneedles + curcumin + ZnO	Dual antibacterial + anti-scarring	ZnO ROS + curcumin TGF-β inhibition	Infection control + scar prevention	animal models	[150]
CT-CS-ZIF@CIP	CS + ZIF-8 + ciprofloxacin	Photothermal + antibiotic release	pH-responsive CIP release + hyperthermia	Antibacterial, healing promotion	animal models	[158]
Fitostimoline^®^ Hydrogel	Triticum vulgare extract (Rigenase^®^) + polyhexanide + glycerine	Plant extract–driven tissue regeneration + antimicrobial protection	Anti-inflammatory modulation (↓ IL-6, TNF-α, NO, PGE2) + fibroblast activation + granulation + autolytic debridement; polyhexanide prevents microbial colonization	Improved local wound signs (pain, erythema, itching); enhanced perilesional skin condition; no safety concerns; no significant difference in complete healing vs. saline gauze	Clinical study: Completed Phase IV RCT (n = 40, 12 weeks)	[159]
GPP@ZnBG Hydrogel	Gel-PBA/PVA network + Zn^2+^ + bioactive glass (Zn, Ca, Si release system)	pH/ROS-responsive sequential ion release for antibacterial + angiogenic repair	Glucose/ROS-triggered bond cleavage → controlled Zn^2+^ burst (antibacterial) → BG degradation releases Zn^2+^/Ca^2+^/SiO_3_^2−^ → HIF-1α/VEGF activation → angiogenesis	Strong antibacterial activity (up to 96%); enhanced angiogenesis; accelerated diabetic wound closure (~99% in mice); pilot clinical study shows ~80% complete closure with good safety	Clinical study: Pilot clinical study (n = 10, 8 weeks); Phase III planned	[160]

Currently, the majority of multifunctional hydrogels are still in the in vitro and animal model stages, with only a limited number progressing to clinical trials. “→” indicates “next step”.

## 4. Outlook

In recent years, hydrogels have achieved notable advances in chronic wound management, with several hydrogel formulations entering clinical trials or receiving approval for applications in surgical sealing and tissue adhesion [161,162]. Nevertheless, their widespread clinical implementation still encounters three major challenges. First, the long-term biosafety of drug-loaded hydrogels necessitates comprehensive systematic evaluation, particularly for therapeutics with hepatotoxic, nephrotoxic, or neurotoxic potential (e.g., cyclosporine and metallic nanomaterials) [156,157,158,159,160,161,162,163]. Second, the preparation processes are complex and costly, and the absence of standardized protocols for efficacy assessment and quality control hinders scalable and reproducible manufacturing [29,44,164,165]. Third, intrinsic differences between animal models and humans limit the translatability of some effective strategies [166,167,168]; Furthermore, interdisciplinary barriers in mechanistic understanding and evaluation often lead to a disconnect between preclinical research and clinical requirements [20,169,170].

In response to these challenges, the future development of multifunctional hydrogels is likely to focus on three principal trends.

### 4.1. Intelligent Closed-Loop Systems and Multidisciplinary Collaboration

With the aid of programmable hydrogel technology [171] and AI-driven big data models [172], hydrogels are evolving from passive dressings into active, responsive therapeutic platforms. At the closed-loop therapeutic level, systems integrating “real-time monitoring, intelligent feedback, and precision treatment” can dynamically detect physiological signals and respond autonomously, thereby markedly enhancing both the precision and timeliness of intervention [172,173]. For instance, the GPP@ZnBG hydrogel responds to hyperglycemia and oxidative stress at the wound site by sequentially releasing zinc, calcium, and silicon ions in a pH-regulated manner, thereby achieving early-stage antibacterial effects and promoting late-stage angiogenesis. Preliminary clinical studies indicate that topical application for four weeks reduced wound area by 94.57%, with no reported adverse events. The tough, adhesive smart hydrogel developed by Shi [174] integrates multiple functionalities, including synergistic photothermal/nitric oxide antibacterial activity, angiogenesis promotion, and signal monitoring, achieving complete wound closure within 14 days. In parallel, the integration of wearable biosensors with smart hydrogels further advances wound management toward closed-loop systems by enabling real-time monitoring of electrical signals (e.g., impedance) and biochemical markers (e.g., pH, glucose, and inflammatory factors), which can trigger responsive interventions such as drug release, electrical stimulation, or photothermal therapy. Collectively, these systems allow coordinated regulation of infection, hypoxia, and inflammation to promote tissue regeneration [138,175,176]; however, they remain limited by insufficient sensing depth, trade-offs between sensitivity and long-term stability, and a lack of clinical validation.

At the research and development level, intensive collaboration among materials science, biology, clinical medicine, and engineering, combined with AI-assisted material design [168] and organoid model validation [177] holds promise for establishing a closed-loop research paradigm encompassing “computational design–high-throughput screening–experimental validation–clinical translation.” Zheng [178] systematically demonstrated how AI can integrate clinical and biological data to predict healing risks, optimize nanocarrier design, and dynamically tailor personalized treatment strategies. Furthermore, stimulus-responsive hydrogels based on cascade reactions have substantially enhanced the rational design and predictive validation of hydrogel systems by integrating AI-assisted modeling, biosensor feedback, and organ-on-a-chip technologies. Collectively, these studies validate the feasibility of closed-loop systems and multidisciplinary collaborative strategies, which are anticipated to substantially accelerate the translational process.

### 4.2. The Deep Integration of Traditional Chinese Medicine Theory and Hydrogel Technology

Beyond addressing the challenges mentioned above, the development of diversified therapeutic strategies represents an inevitable trend. The extensive clinical use of traditional Chinese medicine (TCM) dressings or medicated solutions in diabetic wound management [179,180] provides a solid clinical basis for such integration. Recently, considerable progress has been achieved in integrating bioactive components of TCM with modern smart hydrogel platforms. For example, quercetin, the principal bioactive component of Astragalus membranaceus, loaded onto chitosan nanoparticles within a GelMA hydrogel (QNPs@GelMA), exerts synergistic effects by downregulating the RAGE/NF-κB pathway, thereby providing anti-inflammatory and antioxidant benefits, promoting M2 macrophage polarization and angiogenesis, and accelerating diabetic wound healing [181]. Berberine-loaded glucose-responsive hydrogels (OHA/CMCS-BBR) enable high-glucose-triggered, on-demand drug release through dynamic boronate bonds. In a diabetic infected wound model, these hydrogels achieved a 99.7% wound closure rate within 14 days and completely eradicated bacterial pathogens [182]. A carrier-free hydrogel (Cu-SCU), formed by the self-assembly of scutellarin and copper ions through dynamic coordination bonds, can scavenge reactive oxygen species, induce M2 macrophage polarization, and activate the HIF-1α/VEGF pathway in a diabetic full-thickness wound model, thereby synergistically promoting epithelial regeneration, vascular maturation, and wound repair [183]. Overall, these studies integrate modern materials science with bioactive TCM components, providing an innovative paradigm for the development of multifunctional hydrogels with distinctive TCM characteristics, thereby enabling multi-targeted, synergistic therapy for diabetic wounds.

However, most current studies remain largely limited to single-component integration and have not yet fully realized synergistic design guided by the traditional Chinese medicine “emperor–minister–assistant–courier” (EMAC) compatibility theory [184]. In addition, dose–response relationships of bioactive TCM components remain poorly defined, while their extraction complexity, as well as the physicochemical properties, bioactivity, and potential toxicity of individual constituents, still require systematic characterization [185]. Furthermore, a key challenge lies in balancing the diversity of assembly strategies for incorporating active components within hydrogel matrices against the structural and functional stability of the resulting systems, which remains a critical bottleneck for clinical translation [186].

### 4.3. Standardized Quality Control Systems, Safety Assessment, and Intelligent Manufacturing

To overcome bottlenecks in safety assessment and large-scale production, a standardized quality control system encompassing the entire R&D process is essential. Specifically, drug safety prediction models leveraging organ-on-a-chip technology and computational toxicology should be developed to systematically evaluate pharmacokinetics and accumulation risks following transdermal drug delivery, as well as to establish standardized preclinical safety evaluation guidelines. Concurrently, the integration of automated and continuous production processes can reduce labor and time costs, thereby enabling high-quality, low-cost, large-scale manufacturing. In recent years, several hydrogel-based wound dressings have been translated into clinical use or clinical evaluation, demonstrating their potential for real-world wound management; for instance, INTRASITE* Gel Hydrogel Wound Dressing, DermiSphere hDRT, and the antimicrobial peptide hydrogel Amferia have all received FDA clearance or approval. From an industrial perspective, patent analysis indicates that more than 96,000 hydrogel-related patent documents had been published globally as of September 2024, with approximately 74.5% remaining in the application stage. This reflects a clear shift from single-function materials toward smart, integrated hydrogel platforms [187] (as Table 4). Current patent activity is mainly concentrated in three areas: stimuli-responsive systems, antimicrobial hydrogels, and bioactive delivery platforms [188]. These developments suggest that smart hydrogel dressings are at a critical juncture, transitioning rapidly from laboratory research to clinical application, thereby providing valuable practical guidance for industrial development.

In summary, the future development of multifunctional hydrogels will be driven by three key directions: “smart closed-loop systems and multidisciplinary collaboration,” “deep integration with traditional Chinese medicine,” and “standardized quality control, safety assessment, and intelligent manufacturing.” Among these, the most promising design principle is the construction of multifunctional, stimulus-responsive hydrogels capable of recognizing key pathological cues in diabetic wounds and coordinately regulating inflammation, angiogenesis, and tissue remodeling under clinically relevant thresholds, such as glucose-triggered responses at ~10 mM and pH-responsive activation within the physiological to mildly acidic range (pH 5.5–7.4) [189,190]. From a translational perspective, priority benchmarks should include long-term in vivo degradation of ≥12 months [191], ISO 10993-compliant biocompatibility evaluation, ≥70% complete wound closure within 12 weeks, and ≤20% recurrence within 6 months in randomized controlled trials [192]. However, successful clinical translation will still require standardized preclinical models, clearly defined regulatory pathways, rigorous long-term safety validation, and effective strategies to overcome practical barriers such as high production costs, limited manufacturing infrastructure, storage requirements, and insufficient clinical training resources, particularly in resource-limited healthcare settings [193,194,195].

Addressing these challenges will be essential for facilitating broader clinical adoption and commercialization of multifunctional hydrogels. Overall, this field is shifting toward adaptive, pathology-driven, and clinically translatable hydrogel systems that integrate therapeutic functionality with practical applicability, ultimately advancing precision wound management.

## Figures and Tables

**Table 4:** Summary of representative patents and clinical trials of multifunctional hydrogels for diabetic wound healing.

Category/Region	Identifier/Source	Technology Platform & Mechanism	Therapeutic Functions & Significance	Status & Evidence
Patent/CN	CN121287411A	PEG/PVA/chitosan/alginate + glucose/pH/MMP multi-responsive + BLE/NFC + MCU closed-loop system; integrates 5 sensors + 4 drug release units	Closed-loop monitoring, diagnosis, on-demand multi-drug delivery; represents the highest-level smart dressing concept with integrated sensing-decision-therapy functions	Published 1 September 2026/Patent-level evidence
Patent/CN	CN121338078A (to be verified)	CMCS/OGG dual-crosslinked (Ca^2+^ ionic + Schiff-base) + GS-loaded; pH-responsive release (53% at pH 5.0 vs. 30% at pH 7.4/5 h); injectable + self-healing (G′ recovery, 120 s gelation)	Antibacterial, pH-triggered on-demand release, adaptive wound coverage; swelling ~3800%, >80% degradation at pH 5.0/9 d; applicable to diabetic foot wounds	Published 16 January 2026/Patent-level evidence with quantitative data
Patent/CN	CN119971129A	DEXO/GelMA dual-crosslinked + OCS@MOF@Polyphyllin I; pH-responsive release (67.5% at pH 5.2 vs. 44.3% at pH 7.4/96 h); ~10 s gelation; enzyme/nanomaterial-mediated regulation	Antioxidant (64.69% DPPH), antibacterial (<40% survival), M1 → M2 polarization (CD86 3.07%/CD206 40.5%), angiogenesis promotion; diabetic rat: 94.04% wound closure/14 d	Published 13 May 2025/Preclinical in vivo efficacy
Patent/CN	CN120827634A	Amino acid-crosslinked HHA(400–600 kDa)/LHA(<10 kDa) + OβCD host-guest anchoring + catechol violet AI colorimetric monitoring; pH-programmed M1/M2 immunomodulation (LHA pH 6.0–6.5 → M1; HHA pH 7.0–7.4 → M2)	Long-lasting antibacterial >99% (≥7 d), programmed immune regulation, Li^+^ anti-scarring (vimentin/N-cadherin ↓, E-cadherin/EPCAM ↑), intelligent wound monitoring; diabetic mouse: near-complete healing/14 d	Published 24 October 2025/In vivo efficacy
Clinical Trial/MX (registered in US)	NCT07541196	PAW-Carbopol^®^ 940 + ROS/RNS (DBD plasma); pH 5.5; 2–3×/week; DFU (Wagner 1–2), pressure ulcers (I–III), venous/arterial ulcers; duration > 3 mo, area 2–20 cm^2^	Antimicrobial, anti-inflammatory; RCT (n = 50), 12-week follow-up; primary endpoints: wound area reduction, bacterial load	Recruiting/Level I RCT (double-blind)
Clinical Trial/US (multi-center)	NCT06616844	Porcine placental ECM (PPECM, InnovaMatrix^®^ AC) + collagen/laminin/fibronectin/proteoglycans/TGF-β/VEGF/FGF; ECM-mimetic biological regulation	Tissue regeneration, angiogenesis, ECM reconstruction; specifically targets hard-to-heal DFU	Recruiting/Level I RCT (observer-blinded, n = 50)
Clinical Trial/CN	NCT06492811	GAT@F nanoenzyme (GOx + CAT cascade); GOx: glucose → H_2_O_2_, CAT: H_2_O_2_ → O_2_; once-daily dressing change, 14 days	Glucose consumption, O_2_ generation, ROS regulation; enzyme-responsive smart hydrogel targeting diabetic wound metabolism	Active, not recruiting/Phase II RCT (double-blind, n = 49)
Clinical Trial/US	NCT05607979	Lavior Diabetic Wound Gel vs. Smith & Nephew Solosite Gel; head-to-head non-inferiority comparison	Moist wound healing, DFU management; provides clinical benchmarking data for hydrogel wound products	Completed/Phase II/III RCT (non-inferiority, n = 75)

Data were retrieved from Espacenet (https://worldwide.espacenet.com/) and ClinicalTrials.gov (https://clinicaltrials.gov/) covering the period from 2025 to 2026. “→” indicates “next step”. “n” = “sample size”.

## Data Availability

No new data were created in this study. This article is a review of previously published literature, and all relevant data are available in the cited references.
